# Supplementary material for: Modeling glioblastoma heterogeneity as a dynamic network of cell states
Source: Mol Syst Biol. 2021 Sep 16;17(9):e10105. doi: 10.15252/msb.202010105 (PMC8444284; doi:10.15252/msb.202010105)
Supplement: Supplementary file 6 — Source Data for Figure 5 [file MSB-17-e10105-s004.zip › Figure5A_sourcedata/GSEA_3071/nosigdb_stateIII.GseaPreranked.1622795155562/NB_KI_CLUSTER11.html]

Details for gene set NB\_KI\_CLUSTER11[GSEA]

|  || Dataset | state33071 |
| Phenotype | NoPhenotypeAvailable |
| Upregulated in class | na\_pos |
| GeneSet | NB\_KI\_CLUSTER11 |
| Enrichment Score (ES) | 0.29060233 |
| Normalized Enrichment Score (NES) | 1.5928696 |
| Nominal p-value | 0.048387095 |
| FDR q-value | 0.040510878 |
| FWER p-Value | 0.371 |
Table: GSEA Results Summary

  

Fig 1: Enrichment plot: NB\_KI\_CLUSTER11      
 Profile of the Running ES Score & Positions of GeneSet Members on the Rank Ordered List

  

| PROBE | GENE SYMBOL | GENE\_TITLE | RANK IN GENE LIST | RANK METRIC SCORE | RUNNING ES | CORE ENRICHMENT || 1 | SH3BGRL3 |  |  | 26 | 0.505 | 0.0635 | Yes |
| 2 | PFN1 |  |  | 40 | 0.481 | 0.1270 | Yes |
| 3 | S100A11 |  |  | 48 | 0.464 | 0.1896 | Yes |
| 4 | S100A10 |  |  | 102 | 0.409 | 0.2329 | Yes |
| 5 | S100A6 |  |  | 172 | 0.360 | 0.2652 | Yes |
| 6 | TSPO |  |  | 341 | 0.280 | 0.2612 | Yes |
| 7 | CLIC1 |  |  | 373 | 0.269 | 0.2906 | Yes |
| 8 | LITAF |  |  | 582 | -0.259 | 0.2733 | No |
| 9 | ZC3H12A |  |  | 1024 | -0.281 | 0.1996 | No |
| 10 | TGIF1 |  |  | 1506 | -0.306 | 0.1191 | No |
| 11 | CTSC |  |  | 1511 | -0.306 | 0.1607 | No |
| 12 | NFKBIZ |  |  | 1903 | -0.330 | 0.1065 | No |
| 13 | SLC2A3 |  |  | 2117 | -0.343 | 0.0997 | No |
| 14 | PLAUR |  |  | 2181 | -0.347 | 0.1318 | No |
| 15 | GLIPR1 |  |  | 2390 | -0.364 | 0.1292 | No |
| 16 | FLNA |  |  | 2559 | -0.378 | 0.1388 | No |
| 17 | CCND2 |  |  | 3037 | -0.425 | 0.0758 | No |
| 18 | GYPC |  |  | 3598 | -0.546 | 0.0084 | No |
| 19 | THBS1 |  |  | 3599 | -0.547 | 0.0844 | No |
Table: GSEA details [plain text format]

  

Fig 2: NB\_KI\_CLUSTER11: Random ES distribution      
 Gene set null distribution of ES for **NB\_KI\_CLUSTER11**
